# An Auditory–Vocal Package for Story Recall in Chinese Children with Autism: Evaluating Forward Chaining and Emergent Divergent–Convergent Relations

**DOI:** 10.3390/bs16091483

**Published:** 2026-08-25

**Authors:** Liming Zhou, Xiaoyi Hu, Jiaying Hao, Xinyu Chen, Xiaoya Wu, Yuanyuan Mei, Junyan Chen, Ziqi Xu, Jiamei Zhao, Qi Qi

**Affiliations:** 1 Education Research Center for Children with Autism, Faculty of Education, Beijing Normal University, Beijing 100875, China; lzhou2@comcast.net (L.Z.);; 2 Shanghai Educational Guidance Center for Children with Autism, Shanghai 201999, China; 3 Qingdao Binhai College Special Education Center, Qingdao 266555, China; 4 Beijing Official Portal Management Center, Beijing 100089, China; 5 School of Psychology, Hainan Normal University, Haikou 571158, China

**Keywords:** auditory–vocal arrangement, story recall, verbal behavior, multiple stimulus control, forward chaining, joint control, autism spectrum disorder

## Abstract

This study evaluated the effects of a strictly auditory–vocal instructional package, combining cued vocal rehearsal, forward chaining, and multiple stimulus control arrangements, on the acquisition of forward intraverbal story recall (RC, divergent relations) and the emergent performance on untaught reverse character identification (CI, convergent relations) probes across nine children with autism spectrum disorder (ASD). Utilizing a concurrent multiple-baseline design across character chains replication, the 1:1 intervention integrated the auditory–vocal components without initial permanent visual aids. All nine participants met the mastery criterion for the directly taught forward story-recall chains (mean = 4.6, 4.9, and 4.3 sessions across target characters). Following instruction, unreinforced probe performance on untaught CI relations increased above pre-instruction levels for all participants, with seven achieving mastery on these convergent tasks. Maintenance probes conducted at 2 and 4 weeks post-instruction demonstrated sustained responding for the majority of participants, though marked individual variability—including notable performance decays in story recall for two participants—was observed. Because a formal component analysis was not conducted, the specific contribution of vocal rehearsal cannot be isolated from the overall package, and theoretical mechanisms such as joint control serve only as a cautious, tentative conceptual account. While limited by CI’s functional problem-solving evidence being tempered by its measurement format within the staggered concurrent arrangement, the absence of component isolation, and direct classroom generalization probes, these preliminary findings suggest that package utilizing strictly auditory–vocal arrangements demonstrates initial feasibility for establishing complex intraverbal forward chains under controlled 1:1 clinical conditions. This foundation allows future research on adapting this package, considering temporal location parameter adjustments, to support multi-component stimulus control for vocal independence within inclusive classroom settings like Learning in Regular Classrooms (LRC).

## 1. Introduction

Story recall and narrative comprehension represent foundational academic and social repertoires for children with neurodevelopmental conditions, including autism spectrum disorder (ASD), who are transitioning into inclusive general education classrooms. Establishing rich narrative skills is critical not only for daily social interaction but also for reading comprehension and academic achievement (Spencer & Kirby, 2025). Within the broader educational literature, adapted language interventions have increasingly focused on addressing the unique processing profiles of learners with neurodevelopmental disorders to facilitate functional communication and academic engagement (for a systematic review, see Andreou & Argatzopoulou, 2025).

Within the framework of verbal behavior analysis (Skinner, 1957), retelling a narrative involves emitting an extended intraverbal chain in which successive verbal responses are under the control of a shifting sequence of verbal stimuli (Valentino et al., 2015). Unlike simple or discrete intraverbals (e.g., filling in common phrases), story recall requires a listener to respond to compound verbal stimuli that are inherently transient, long-form, and rapidly presented. In typically developing children, narrative proficiency often co-occurs with self-directed verbal behaviors, such as vocal rehearsal, which may assist in bridging delays between an auditory antecedent and a subsequent opportunity to respond. However, this task poses substantial challenges for children with ASD, who frequently exhibit difficulties in responding to complex intraverbal tasks under multiple stimulus control (Axe, 2008; Sundberg & Partington, 1998; Sundberg & Sundberg, 2011).

Recent behavior-analytic research has systematically addressed story retelling using prompt fading and chaining procedures combined with various prompt modalities. For example, de Matos et al. (2017) evaluated a prompt-fading protocol incorporating textual and echoic scaffolds to teach narrative sequences. Conversely, Valentino et al. (2015) and Conine et al. (2023) utilized modified backward-chaining procedures supplemented by pictorial and textual supports. Despite the documented efficacy of these interventions, a notable feature of prior research is the heavy reliance on external visual supports that must be systematically faded to achieve independent vocal responding (Conine et al., 2023; Valentino et al., 2015). Although visual supports provide immense benefit to many autistic learners, auxiliary visual scaffolding can sometimes be challenging to individualize, manage, or fade within standard public school classrooms.

This practical challenge is particularly relevant within China’s inclusive education model, known as “Learning in Regular Classrooms” (LRC. Under the LRC framework, standard classrooms are characterized by large group sizes (sometimes exceeding 40 students) and high student-to-teacher ratios. In these fast-paced settings, instructional delivery is overwhelmingly auditory-reliant, making it difficult for general education teachers to continuously manage individual physical or visual prompt-fading protocols. Furthermore, continuous reliance on external visual stimuli carries a potential risk of prompt dependency, which may restrict the sustainability of narrative interventions in natural group environments. Therefore, evaluating procedural extensions that establish complex intraverbal repertoires through auditory–vocal arrangements—rather than initiating instruction with heavy visual scripts—warrants empirical investigation to explore cross-setting utility within existing classroom structures.

A parsimonious approach to auditory–vocal arrangements involves transferring stimulus control from external auditory models to cued, self-generated verbal responses that function alongside the target chain (Cariveau et al., 2022). Extended verbal sequences are often accompanied by precurrent behaviors—such as immediate echoic or self-directed vocalizations—that occur immediately following the presentation of a transient verbal stimulus. In narrative recall, a learner may engage in vocal rehearsal to replicate the acoustic features of an instructor’s narration, assisting in bridging the temporal gap between the antecedent story and the subsequent intraverbal query. This self-directed verbal behavior may be maintained by a combination of automatic reinforcement—where the learner’s vocalizations achieve parity with the auditory model (Palmer, 1996)—and intermittent social reinforcement. Establishing these cued rehearsal responses may alter the functions of subsequent stimuli, possibly assisting in the acquisition of extended intraverbal chains without relying on permanent external visual scaffolds (Palmer, 1991).

From a conceptual perspective, the role of cued vocal rehearsal during intraverbal instruction may also be tentatively examined alongside behavior-analytic accounts of multiple stimulus control and Lowenkron’s (1998) analysis of joint control (Zhou et al., 2025). Within joint control paradigms, a cued verbal response maintained through active repetition co-occurs alongside a second stimulus to evoke a specific response class, an arrangement that has been evaluated to establish complex conditional discriminations in individuals with developmental delays (Tu, 2006). However, whether an instructional package incorporating vocal rehearsal can support performance on untrained reverse tasks—such as moving from forward story recall (“What did the character do?”) to reverse character identification (“Who performed these actions?”)—warrants careful empirical examination (Cariveau et al., 2022). Evaluating whether an instructional package utilizing vocal rehearsal accompanies the emergence of untrained reverse responses may offer tentative, preliminary insights into how functional problem-solving skills develop within the verbal repertoires of autistic learners (Skinner, 1968).

The present study was designed as a procedural extension of the intraverbal chaining frameworks established by Valentino et al. (2015) and Conine et al. (2023). While prior research relied on external visual scripts requiring systematic fading, this study evaluated the preliminary efficacy of a multi-component instructional package—utilizing cued vocal rehearsal within a cumulative forward-chaining framework without initiating training with external visual prompts—on the acquisition of forward story recall and performance on untrained reverse character-identification probes. By shifting to a purely auditory–vocal arrangement, this study attempted to address a practical gap relevant to group-classroom contexts where fading physical cues is restricted. Grounded in an applied verbal behavior framework (Skinner, 1957), this study aimed to: (1) evaluate the efficacy of the multi-component package in establishing complex intraverbal chains; (2) examine whether training story recall via the instructional package would be associated with emergent performance on untrained reverse character-identification probes; and (3) observe the persistence of these verbal behaviors during post-instruction follow-up probes. Ultimately, this approach seeks to evaluate an adaptable instructional arrangement that supports verbal independence in inclusive settings.

## 2. Method

### 2.1. Participants and Setting

Nine children (2 females, 7 males) diagnosed with autism spectrum disorder (ASD) according to DSM-5 criteria via multiple clinical teams participated in this study. Their chronological ages ranged from 5 years 2 months to 9 years 7 months, and their scores on the Wechsler Intelligence Scale for Children (WISC) demonstrated a wide cognitive range (IQ = 40–114; Table 1). Participants met the following inclusion criteria: (a) a total score above 100 on the Chinese version of the Verbal Behavior Milestones Assessment and Placement Program (VB-MAPP), demonstrating entry-level proficiency in Level 3 milestones (Sundberg, 2014); (b) an established vocal echoic repertoire, including the ability to echo multi-syllable combinations (3–5 syllables) and simple sentences; (c) an expressive vocabulary exceeding 1200 words based on standard measures (Zhang & Li, 2008); and (d) documented observations of spontaneous, overt self-echoic behaviors during typical academic routines.

The broad range of chronological ages and IQ scores across participants reflects a heterogeneous sample profile. This variation represents a relevant descriptive variable, as differences in cognitive or foundational verbal repertoires may correspond with variations in learning velocity or performance on untaught reverse tasks. In single-case experimental methodology, this individual heterogeneity is systematically managed by utilizing each participant as their own internal experimental control across dense, repeated measurement conditions.

Prior to instruction, stable baseline performance was established across all targeted verbal chains for each participant. Specifically, all participants demonstrated zero levels of independent story recall (0.0% accuracy) during baseline. On pre-instruction probes evaluating untaught reverse character identification, scores averaged 14.8% across participants (*range = 0.0–33.3%*), establishing stable baseline trends prior to intervention. The sequential introduction of the multi-component instructional package across the distinct intraverbal character chains—systematically replicated across the nine individual participant profiles within a concurrent multiple-baseline design—allows for the observation of whether behavioral changes correspond directly with the onset of instruction, irrespective of baseline variations in participant age or IQ.

### 2.2. Setting and Implementers

All instructional and probe sessions were conducted individually in quiet, designated areas within standard classrooms (approximately 6 m × 6 m) across three cities in China. To eliminate potential visual scaffolds and systematically evaluate performance under purely auditory–vocal arrangements, no printed texts, picture books, or visual illustrations were present within the immediate instructional environment during baseline, instructional, or probe trials.

The implementation team consisted of board-certified behavior analysts and special education teachers, all of whom had over five years of professional experience in applied behavior analysis and developmental disabilities. Although all implementers underwent comprehensive training on the protocol before the study began, and strict procedural fidelity checklists were maintained across all sessions to ensure delivery consistency, these conditions represented a highly controlled 1:1 instructional arrangement delivered by experienced clinicians rather than standard group-classroom routine delivery by general education teachers.

### 2.3. Materials and Stimuli

The instructional stimuli consisted of an adapted narrative titled “Three Friends”, Appendix A, derived from supplementary reading exemplars within the national Chinese language curriculum standards for compulsory education. The narrative was selected cooperatively by the experimenters and the participants’ primary language instructors to ensure that its vocabulary, syntax, and overall linguistic complexity were aligned with the baseline verbal repertoires of the participants. The narrative featured three distinct characters (Ant, Fox, and Turtle) interacting with a common focal element (a pile of grain). To construct the targeted intraverbal chains, each character was systematically associated with a sequence of four actions, which functioned as transient verbal antecedents across the narrative progression (Table 2).

### 2.4. Experimental Design and Dependent Variables

A concurrent multiple-baseline design across behaviors—specifically targeting three distinct intraverbal chains associated with each story character (Ant, Fox, and Turtle)—was utilized to evaluate the effects of the multi-component instructional package. The introduction of the intervention was staggered systematically across the three intraverbal chains to demonstrate experimental control, with this concurrent arrangement replicated across all nine participants.

The primary dependent variable was story recall, defined as the number of target intraverbal segments emitted independently within 5 s following the instructor’s vocal question, “What did [character] do?” To be scored as correct, the participant was required to vocally recall the target four-action sequence associated with that specific character without external prompts. While this scoring method adapted the structural criteria outlined by Valentino et al. (2015), the current study utilized a cumulative forward-chaining arrangement rather than a modified backward-chaining protocol.

The secondary dependent variable was untaught character identification, evaluating performance on reverse relations. This variable was defined as the number of correct character names emitted within 5 s following a compound vocal question presenting the four sequential actions in reverse (e.g., “Who asked about the grain, plowed, raced, and harvested?”). This reverse relation was never directly trained or reinforced during instruction; it was evaluated solely via unreinforced probes to observe whether the acquisition of forward intraverbal chains was accompanied by performance on untaught reverse character identification.

Responses for both dependent variables were scored using a binary system (correct/incorrect). For story recall, each of the four sequential actions was scored individually as well as collectively for the full forward intraverbal chain. An individual segment was scored as correct only if the participant emitted the target action independently within 5 s of the query. Conversely, omissions, factual errors, perseverations, or prompted responses (i.e., those occurring after any instructor prompt or during error correction) were scored as incorrect. This dual-scoring approach allowed for both an aggregate accuracy score per character chain (0.0–100.0%) and a descriptive analysis to evaluate specific response patterns and structural disruptions within the verbal chain.

Descriptive analysis of individual participant performance on unreinforced probes for untaught reverse character identification (CI) revealed marked individual variations in responding. While a descriptive metric to quantify non-overlap was considered, we subsequently determined that the limited measurement topography of CI probes within this simultaneous staggered arrangement is insufficient to support robust statistical parameters. Therefore, the observed individual difference heterogeneity in CI accuracy, characterized by variable performance decaying during temporal delays for some participants, suggests that complex auditory–auditory compound arrangements may lead to Restricted Stimulus Control or overshadowing (Cariveau et al., 2022). These individual differences in CI performance modulated the functional interpretation of the emergent relation, warranting future research to systematically isolate temporal location parameters within multi-component auditory arrangements (Cariveau et al., 2022).

### 2.5. Interobserver Agreement and Procedural Fidelity

All baseline, instruction, and probe sessions were video-recorded. A secondary independent observer reviewed approximately 33.3% of randomly selected sessions balanced across all experimental conditions and participants. Interobserver agreement (IOA) was calculated using a segment-by-segment agreement formula (agreements divided by the sum of agreements and disagreements, multiplied by 100%). The mean IOA for participant responses across all probe and instructional sessions was 98.9% (*range = 85.0–100.0%*).

Procedural fidelity was assessed via a structured checklist verifying the accurate delivery of task discriminative stimuli, prompt-fading delays, token reinforcement delivery, and error-correction protocols. Mean procedural fidelity was 98.0% (*range = 95.0–100.0%*) during instructional sessions and 94.0% (*range = 87.0–100.0%*) during probe sessions. The lower bounds of procedural fidelity observed during certain probe sessions (87.0%) were exclusively associated with minor, non-critical procedural variations, such as marginal latencies in question delivery or pacing adjustments corresponding to participant non-compliance. In no instances did these variations involve the accidental delivery of instructional prompts or social reinforcement, thereby maintaining strict procedural distinctions between baseline, instructional, and probe conditions.

### 2.6. Social Validity

A total of 35 Chinese language teachers with experience in autism education and 20 family members evaluated the social validity of the intervention. After viewing three randomly selected instructional video clips, evaluators completed a 6-item, 5-point Likert scale (1 = Strongly Disagree, 5 = Strongly Agree). This social validity questionnaire was developed by the experimenters specifically for this study to address dimensions relevant to language training in inclusive settings and was administered anonymously; as such, it represents an unvalidated instrument. The scale assessed: (a) the social importance of story-recall skills; (b) general satisfaction with the instructional package; (c) the acceptability and feasibility of procedural steps; (d) the cultural alignment of the package with Chinese pedagogical practices; (e) perceived efficacy for skill retention; and (f) perceived utility in fostering verbal independence. All Likert scale data were aggregated and analyzed descriptively, calculating overall and item-level mean scores, standard deviations (SD), and response ranges (Table 2).

### 2.7. General Arrangements

Prior to each session, including both instruction and probe trials, the instructor obtained the participant’s vocal assent by stating: “We are going to learn an interesting story together. Do you mind if I tell you the story and ask some questions? If you know the answer, please tell me; if not, you can stay quiet or say so. You can stop anytime if you want.” Sessions were postponed or terminated immediately if a participant exhibited distress or declined to participate.

**Figure 1:**
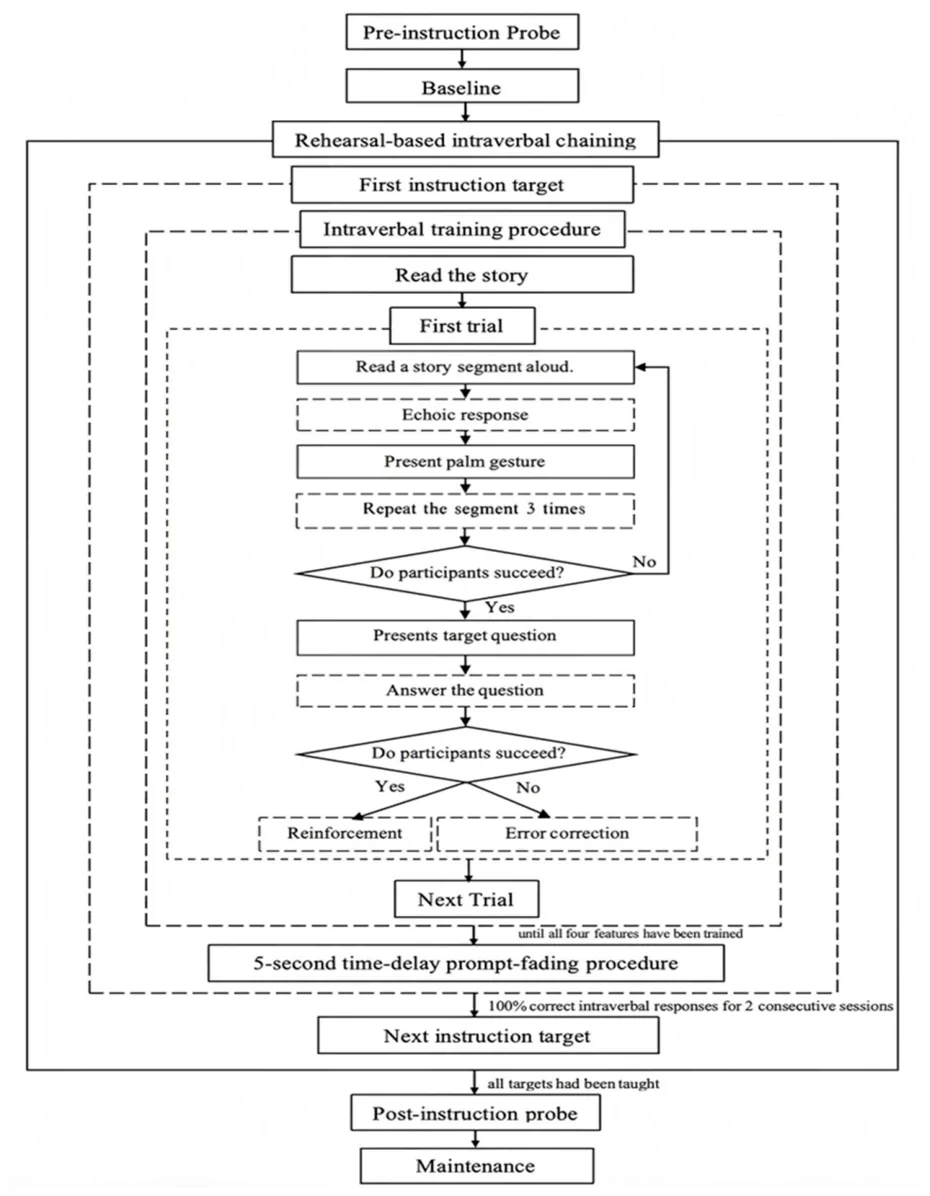
Sequence of pre-instruction probe, pre-probe, baseline, instruction, post-instruction probe, and maintenance.

All trials were conducted in a controlled 1:1 instructional arrangement within a designated area of the standard classroom. To maintain a purely auditory–vocal arrangement and prevent confounding effects from auxiliary stimuli, no printed text, pictorial sequences, or other permanent visual supports were accessible to participants within the immediate environment during baseline, instructional, or probe conditions. A schematic representation of the experimental sequence is detailed in the procedural flowchart (Figure 1).

### 2.8. Pre-Instruction and Post-Instruction Probes

Pre-instruction probes were conducted one week prior to the baseline phase to establish initial performance boundaries for both directly taught forward story recall and untaught reverse character identification. On pre-instruction probes, story recall was 0.0% across all participants, whereas character identification averaged 14.8% (range = 0.0–33.3%), with several participants displaying non-zero baseline levels.

Post-instruction probes were originally scheduled to occur 1 week following the completion of the final instructional phase to evaluate subsequent performance and untaught reverse relations. However, due to administrative scheduling variations within the school facilities, post-instruction probes for Participants 1, 2, 3, and 4 occurred two weeks after they met the mastery criterion. This protocol adjustment resulted in a one-week delay for these four participants, compared to the standard one-week interval maintained for the remaining five participants (P5–P9). The descriptive implications of this temporal variation on their probe performance are evaluated within Section 4.

During story-recall probes, the instructor read the corresponding narrative context and delivered the vocal discriminative stimulus, “What did [character] do?” A total of three randomized trials (one per character target) were completed during each probe session. During character-identification probes, the instructor presented the compound four-action sequence followed by the query, “Who [action 1, 2, 3, and 4]?” No programmed reinforcement, tokens, or corrective feedback were delivered contingent upon response accuracy during probe sessions; however, non-contingent social praise was provided on an intermittent schedule to support general task engagement.

### 2.9. Baseline

Baseline sessions were structurally identical to the story-recall probes. During each baseline session, the instructor read the corresponding narrative context and delivered the vocal discriminative stimulus across three randomized trials (one per character target) within each participant’s repertoire. This was conducted to evaluate the baseline level of independent verbal responding across all three distinct intraverbal chains and to establish stability before the staggered introduction of the multi-component intervention to a specific target chain. Instructors provided non-contingent social praise for general cooperative participation on an intermittent schedule, independent of response accuracy.

### 2.10. Pre-Training: Rehearsal Cued Gesture Condition

Prior to the implementation of the intraverbal chaining procedure, participants were trained to execute an overt vocal rehearsal routine in response to a visual gesture (a raised palm), which functioned as a discriminative stimulus (SD) for overt repetition. The instructor vocally modeled a target sentence from the narrative while presenting the raised palm gesture. Correct echoic responses emitted within 5 s were reinforced with social praise. The instructor then presented the visual gesture paired with the vocal prompt “Repeat,” systematically fading the vocal prompt until the presentation of the visual gesture independently and reliably evoked the vocal repetition of the verbal sequence. This pre-training phase continued until the participant achieved the mastery criterion of independent execution across two consecutive sessions, supporting behavioral readiness for the subsequent multi-component instructional package.

### 2.11. Rehearsal-Based Intraverbal Chaining Instruction

The multi-component instructional package adapted the intraverbal chaining framework described by Valentino et al. (2015) by utilizing a cumulative forward-chaining arrangement (rather than a modified backward-chaining protocol) and substituting external visual scaffolds with cued overt rehearsal routines. In accordance with forward-chaining arrangements, the four-action sequence for each character was partitioned into discrete verbal links and taught cumulatively. Instructional trials were delivered systematically for the target intraverbal chain during each session until the mastery criterion was reached. A 0 s prompt delay was utilized during the initial instructional session for any newly introduced component link (wherein the prompt in Step 1 was delivered immediately following the task demand), followed by a 5 s constant time-delay (CTD) prompt-fading procedure during subsequent sessions.

A standard instructional trial proceeded according to the following five-step sequence:

**Step 1: Auditory Antecedent and Prompt Window**. The instructor read the narrative context and delivered the primary task discriminative stimulus (SD) (e.g., “What did the Ant do?”). During CTD sessions, if the participant independently emitted the currently trained sequence within a 5 s window, the trial advanced directly to Step 5 (Reinforcement). If response latency exceeded 5 s or an error occurred (or during 0 s delay sessions), the instructor immediately delivered an explicit vocal echoic prompt of the targeted component (e.g., “The Ant plowed and watered”).

**Step 2: Echoic Response**. The participant emitted a vocal echoic repetition of the prompted segment within 5 s of the instructor’s model.

**Step 3: Vocal Rehearsal Loop**. Following the correct echoic response, the instructor presented the pre-trained raised palm gesture (SD) to evoke vocal repetitions of the segment three times, systematically stabilizing the verbal sequence through overt rehearsal.

**Step 4: Intraverbal Re-Presentation**. The instructor re-presented the primary vocal discriminative stimulus (SD) (e.g., “What did the Ant do?”), requiring the vocal emission of the currently trained sequence within a 5 s response window.

**Step 5: Reinforcement or Error Correction**. Independent responses emitted during Step 1 produced high-magnitude behavior-specific praise and a tangible token. Correct responses following the rehearsal loop in Step 4 produced only brief social praise to maintain a differential reinforcement schedule. If the response in Step 4 was incorrect or exceeded 5 s, the instructor immediately implemented an error-correction protocol consisting of a full vocal echoic model, the cued rehearsal requirement, and a final re-presentation of the primary intraverbal query.

Mastery of an individual segment required 100% independent accuracy—defined as correct responses occurring exclusively during Step 1 prior to the delivery of any prompt—across two consecutive sessions, after which the next component was added to the chain. Completion of the entire instructional package required the participant to independently recall all four target actions in the correct sequential order without prompts for two consecutive sessions.

### 2.12. Maintenance Probes

Follow-up maintenance probes were conducted at 2- and 4-week intervals following the mastery of all three targeted intraverbal chains. These probes duplicated the structural and non-reinforcement parameters of the post-instruction probe sessions, evaluating both the durability of directly taught forward story recall and the persistence of untaught reverse character-identification relations.

## 3. Results

### 3.1. Acquisition of Directly Taught Story Recall

**Figure 2:**
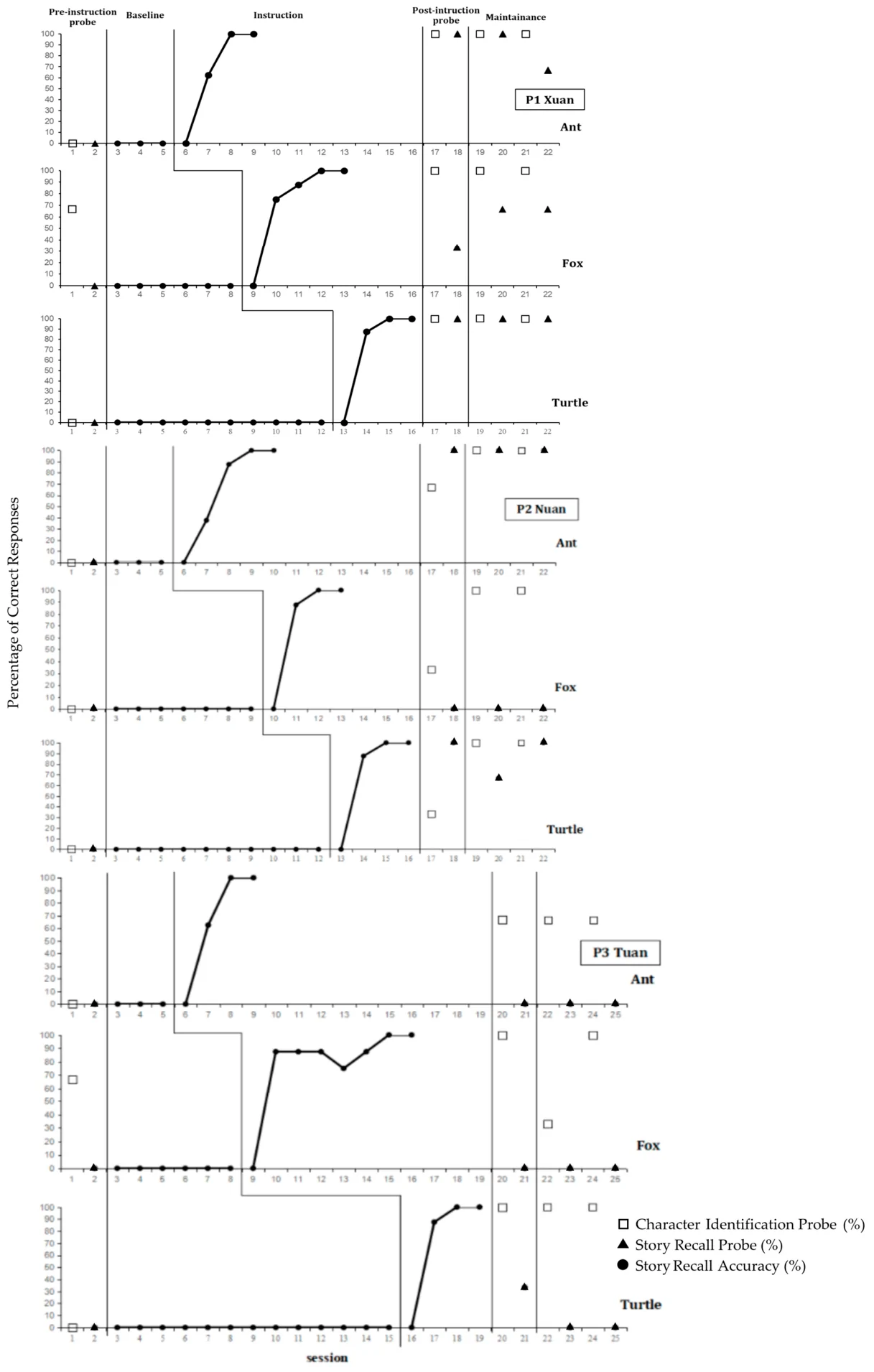
Percentage of independent correct responding during story recall and character identification for P1 (top tier), P2 (middle tier), and P3 (bottom tier) across baseline, instruction, and follow-up phases.

**Figure 3:**
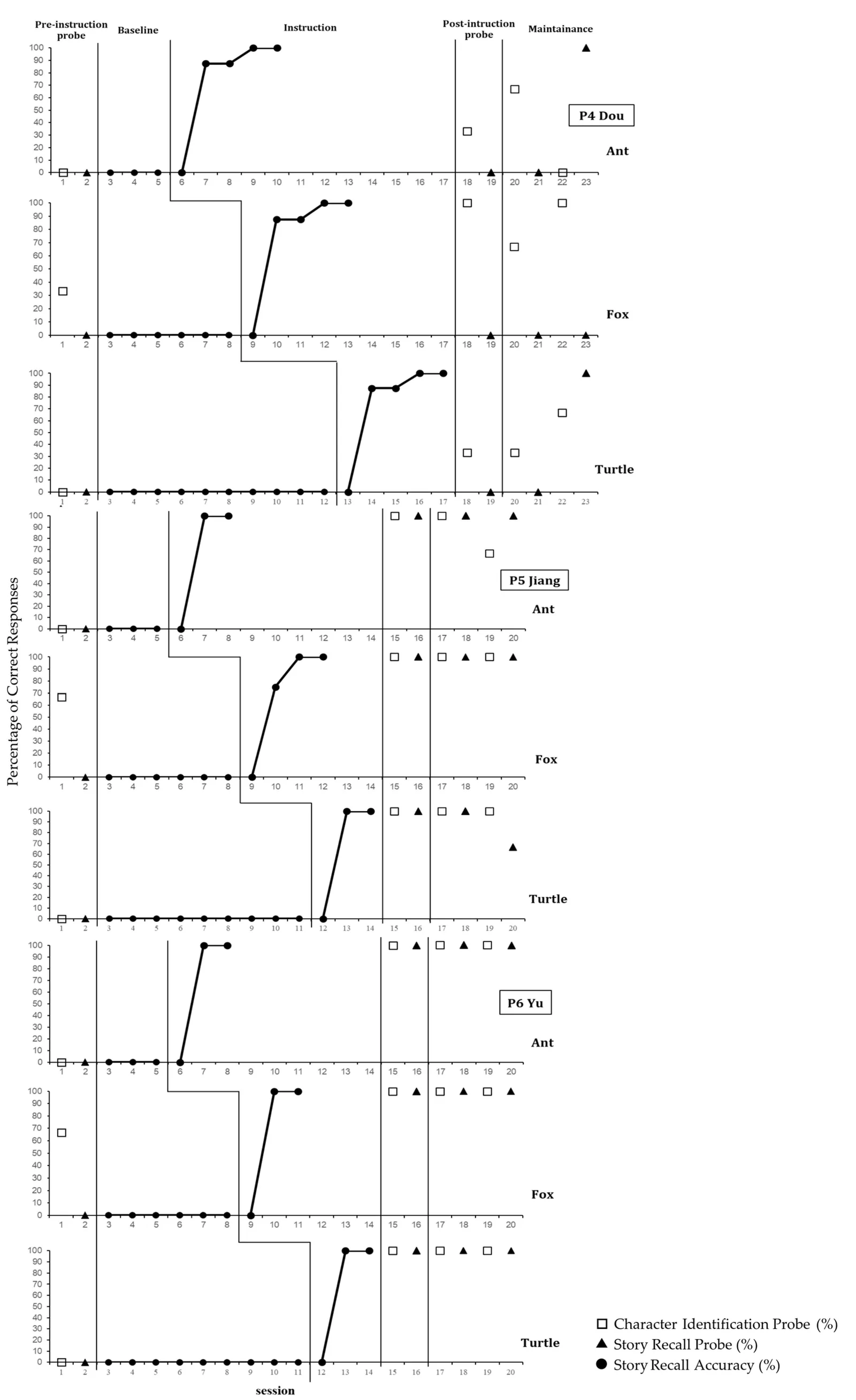
Percentage of independent correct responding during story recall and character identification for P4 (top tier), P5 (middle tier), and P6 (bottom tier) across baseline, instruction, and follow-up phases.

**Figure 4:**
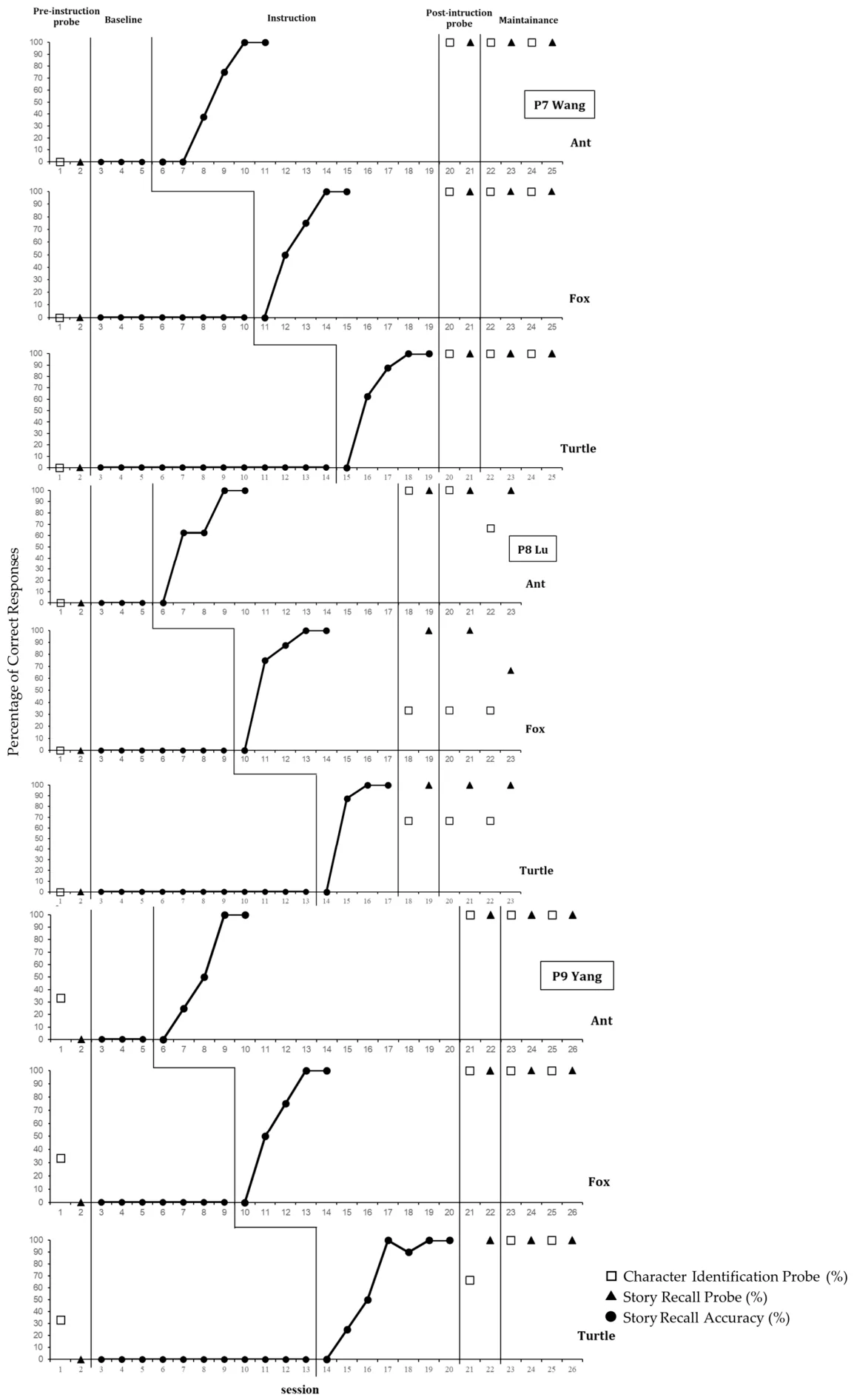
Percentage of independent correct responding during story recall and character identification for P7 (top tier), P8 (middle tier), and P9 (bottom tier) across baseline, instruction, and follow-up phases.

During the baseline phase, all nine participants demonstrated zero levels of independent story recall (0.0% accuracy) across all three targeted character chains, emitting no correct four-action intraverbal sequences (Table 3; Figure 2, Figure 3 and Figure 4). Following the systematic implementation of the multi-component instructional package, an immediate and substantial increase in independent story recall was observed across all participants, with all nine individuals subsequently meeting the mastery criterion of 100.0% independent accuracy across two consecutive sessions. The instructional package demonstrated high efficiency, requiring a mean of 4.6 sessions for Ant, 4.9 sessions for Fox, and 4.3 sessions for Turtle (overall range = 3–8 sessions) for participants to master the complex verbal chains (Table 3).

A distinct variation in initial post-instruction probe performance was observed across participants, coinciding with differences in assessment scheduling timelines. Participants 5, 6, 7, 8, and 9, who received their post-instruction probes according to the standard timeline following mastery, maintained high levels of accuracy (100.0% for story recall; 66.7–100.0% for character identification). Conversely, Participants 1, 2, 3, and 4 exhibited a temporary reduction in independent story recall during their initial post-instruction probe session (range = 0.0–77.8%). As noted in the procedural description, these four participants experienced a protocol adjustment resulting in a two-week post-mastery gap prior to the probe due to external school scheduling conflicts, compared to the standard one-week interval for P5–P9. This lower performance coincided with the extended temporal interval during which active instruction and dense schedules of programmed reinforcement were suspended (Table 3).

### 3.2. Performance on Untaught Character Identification

Prior to instruction, performance on pre-instruction probes for untaught reverse intraverbal relations (character identification) averaged 14.8% across participants (range = 0.0–33.3%), with several participants (P1, P3, P5, P6, P9) displaying non-zero baseline levels on these probes. Following the mastery of the forward story-recall chains, correct character identification on post-instruction probes increased to a mean of 82.7% (range = 44.4–100.0%) across all nine participants, despite these reverse relations never being directly trained or reinforced.

A descriptive observation of individual performance indicated variation that corresponded with participant engagement and procedural compliance during instruction. Participants who consistently executed the instructional steps (e.g., P1, P5, P6, and P7) displayed high accuracy (88.9–100.0%) on the untaught character-identification probes. In contrast, participants who demonstrated lower engagement or variable execution during instructional trials (e.g., P2 and P4) exhibited lower performance on the reverse character-identification probes, scoring 44.4% and 55.6%, respectively (Table 3; Figure 2, Figure 3 and Figure 4).

### 3.3. Maintenance and Follow-Up Performance

Follow-up maintenance probes conducted at 2- and 4-week intervals post-instruction revealed marked individual variability across participants rather than uniform skill retention. Overall, participants demonstrated a mean accuracy of 71.6% (*range = 0.0–100.0%*) at 2 weeks and 75.3% (*range = 0.0–100.0%*) at 4 weeks for forward story recall. For reverse character identification, overall mean accuracy remained relatively stable at 87.7% (*range = 55.6–100.0%*) at 2 weeks and 88.9% (*range = 55.6–100.0%*) at 4 weeks.

Crucially, disaggregated individual response patterns revealed substantial heterogeneity across participants, with severe performance decays observed in specific individuals for forward story recall (SR). Most notably, Participant 3 (P3) exhibited a complete decay in forward story recall, dropping to 0.0% accuracy at both 2-week and 4-week maintenance probes (while maintaining character identification at 66.7% and 88.9%, respectively). Similarly, Participant 4 (P4) scored 0.0% on story recall at the 2-week follow-up before displaying partial recovery to 55.6% at 4 weeks. Participant 2 also maintained reduced story-recall accuracy (55.6% at 2 weeks and 66.7% at 4 weeks). In contrast, Participants 1, 5, 6, 7, 8, and 9 maintained high accuracy levels (77.8–100.0%) across follow-up intervals, with P5 showing only a minor decline at 4 weeks (88.9%) compared to 2 weeks (100.0%). These individual variations occurred across extended gaps without the reintroduction of active instruction or programmed reinforcement (Table 3; Figure 2, Figure 3 and Figure 4).

### 3.4. Descriptive Collateral Reports

In addition to the primary behavior-analytic metrics, descriptive logs from classroom teachers and family members indicated that six of the nine participants were observed to independently emit the vocal rehearsal sequence during novel, non-targeted academic tasks within general education settings. These collateral observations describe untaught occurrences of the vocal rehearsal routine outside of the specific instructional context, indicating that the taught verbal sequence occurred alongside novel academic tasks encountered in both school and home environments. However, because these observations were collected via secondary qualitative logs without systematic experimental manipulation or control conditions, they are interpreted strictly as descriptive collateral outcomes rather than experimentally demonstrated functional generalization.

#### Social Validity

The descriptive data derived from the social validity questionnaires indicated favorable acceptability regarding the intervention’s goals, procedures, and observed outcomes among both educational practitioners and family members (Table 4). As detailed in the summary table, teachers assigned high ratings to the package’s cultural alignment with Chinese pedagogical norms (Item 4; *M = 4.62*, *SD = 0.49*). Respondents noted that the rehearsal-based structure parallels traditional vocal recitation practices, describing it as a familiar instructional modality within Chinese general education classrooms. Furthermore, educators rated the acceptability and feasibility of the procedural steps favorably (Item 3; *M = 4.54*, *SD = 0.51*), noting that the reliance on auditory–vocal arrangements supported the implementation of the package in inclusive environments where specialized external visual aids are often less accessible. Teachers also reported positive evaluations regarding the strategy’s utility in supporting verbal independence (Item 6; *M = 4.40*, *SD = 0.55*).

Family members reported a corresponding level of satisfaction. Parents highlighted the social importance of story-recall skills (Item 1; *M = 4.80*, *SD = 0.41*), reporting that narrative recall and character identification are relevant for participation in family and community functions. Additionally, parents identified the perceived efficacy for skill retention (Item 5; *M = 4.45*, *SD = 0.50*) as a relevant factor in their evaluation, observing that their children mastered the verbal sequences within the instructional timeframes.

In the open-ended feedback section, several educators described this instructional approach as a potential alternative to external visual scaffolding. Practitioners noted that as participants practiced the overt self-rehearsal routine, they observed brief increases in independence during subsequent classroom listening tasks. These observations were consistent with anecdotal parental feedback; for instance, one parent reported that their child no longer required picture cards to recount daily school occurrences, noting that the practice of repeating the words out loud appeared to support independent spoken retention in home contexts.

## 4. Discussion

This preliminary, proof-of-concept study evaluated the effects of a multi-component auditory–vocal instructional package—combining cued vocal rehearsal, cumulative forward chaining, time-delay prompt fading, and programmed reinforcement—on establishing forward intraverbal story recall and evaluating performance on untaught reverse character-identification tasks across nine Chinese-speaking children with ASD. The outcomes indicate that systematic implementation of the multi-component package was associated with an increase in intraverbal narrative responses. Because a formal component analysis was not conducted, the specific contribution of the cued vocal rehearsal routine cannot be isolated from the overall instructional package. Rather than evaluating a novel theoretical account of internal verbal relations, these findings describe a procedural variation and practical application of the intraverbal chaining arrangements described by Valentino et al. (2015) and Conine et al. (2023). By omitting external visual prompts or textual scaffolds, this work demonstrates the preliminary feasibility of utilizing auditory–vocal arrangements in a 1:1 instructional context, offering a foundation for future research in inclusive educational environments.

### 4.1. A Conceptual Account of Story Recall and Behavioral Emergence

The instructional efficiency and subsequent skill acquisition observed in this study may be conceptually examined through behavior-analytic accounts of the verbal mediation of behavior (e.g., Palmer, 1991; Schlinger & Blakely, 1987, 2026). Within this interpretive framework, the explicit rehearsal requirement is conceptualized as part of a cued verbal chain within a multi-component package, rather than an isolated cognitive retrieval mechanism. The variation in performance on untaught reverse tasks can be examined cautiously relative to behavioral accounts of verbal mediation.

Responding to a novel question when the original narrative stimulus is no longer present in the immediate environment describes a complex instance of verbal behavior. Under these conditions, the cued vocal rehearsal routine established in this package may operate as a localized precurrent response. By emitting these vocal topographies, the learner generates an array of supplementary vocal discriminative stimuli (SD). The cumulative effect of these self-generated vocal stimuli may alter the probability of subsequent intraverbal linkages, making the target story-recall chain more probable relative to competing verbal responses. It is possible that the participant’s overt vocal rehearsal operated as an intermediate response that co-occurred with the transient acoustic stimulus across brief delays.

Furthermore, extended intraverbal recall requires ordering individual verbal units into syntactically structured sequences. As learners acquire these multi-unit intraverbal chains, the verbal responses are likely constrained and organized by generalized autoclitic frames (Skinner, 1957). These structural frames (e.g., “[Character] + [Action] + [Location]”) provide a foundational relational template, allowing newly acquired intraverbal terms to be sequenced correctly during narrative recall.

The maintenance and emission of these complex verbal topographies may also be supported by multiple sources of reinforcement. Beyond the explicit generalized conditioned reinforcers (e.g., praise) delivered by the instructor, automatic reinforcement may play a functional role. Specifically, as the child’s vocal responses increasingly match the acoustic models provided during instruction, the self-generated auditory feedback may achieve parity with the target verbal model (Palmer, 1996; Vocal Parity Theory). Achieving parity serves as an intrinsic source of automatic reinforcement that can strengthen and sustain vocal rehearsal independent of immediate social delivery. Combined with intermittent social approval from adults, these co-occurring reinforcement processes likely support both the acquisition and extended retention of narrative intraverbal repertoires.

However, because internal mediational processes, autoclitic framing, and automatic reinforcement mechanics were not directly isolated or manipulated, this descriptive account remains a tentative conceptual interpretation.

### 4.2. Multi-Component Stimulus Control as a Tentative Interpretive Framework

The observed performance on the untaught reverse tasks is consistent with a descriptive account of multiple stimulus control, though alternative configurations must be considered. Rather than asserting the operation of unobservable internal mechanisms, Lowenkron’s (1998) conceptual framework of joint control is utilized strictly as a tentative interpretive guide to analyze observable verbal performance, rather than as a definitive demonstration of an underlying behavioral process (Aragon et al., 2024; Jennings et al., 2024; Brown et al., 2024; Zhou et al., 2025).

During the reverse intraverbal probes (character recognition), participants were presented with a compound acoustic sequence (e.g., “Who plowed, raced…?”). It is possible that the participant’s vocal rehearsal sequence co-occurred with the presentation of this antecedent stimulus, describing a potential arrangement where two distinct sources of antecedent stimulus control converged to evoke the character’s name. However, because these potential mediational verbal links were not directly manipulated or isolated experimentally, this arrangement remains purely interpretive. The observed performance variations across participants cannot rule out alternative accounts of environmental stimulus control, such as chained intraverbal responding, sequential stimulus control, partial stimulus control, or general rote chain completion strategies.

### 4.3. Instructional Efficiency and Ecological

#### Validity Considerations

From an applied perspective, the multi-component package demonstrated favorable instructional efficiency, with participants achieving the mastery criterion within a mean of 4.6, 4.9, and 4.3 sessions across target characters. This rate of acquisition compares favorably with temporal parameters described by Conine et al. (2023), which is notable given the auditory stimulus control arrangements imposed by an auditory–vocal modality.

By omitting external visual scaffolds, the current instructional package describes an arrangement that reduces prompt dependency on physical materials. However, critical caveats regarding ecological validity must be emphasized. This intervention was conducted strictly within highly controlled 1:1 clinical and special education arrangements delivered by experienced behavior-analytic practitioners. It was not evaluated within large, general education classrooms (e.g., classes of 40+ students typical in mainstream Chinese schools) nor was it delivered by general education teachers. Therefore, claims regarding the immediate applicability or ecological validity of this package within mainstream inclusive settings, such as Learning in Regular Classrooms (LRC), cannot be made. Instead, these findings establish only the preliminary procedural feasibility of the package under 1:1 conditions. Future research is required to evaluate whether and how these auditory–vocal procedures can be adapted for implementation by classroom teachers in “Learning in Regular Classrooms” (LRC)-inclusive environments.

### 4.4. Individual Variations in Rehearsal

#### Consistency and Response Maintenance

A disaggregated analysis of individual participant performance suggests that variations on untaught reverse tasks corresponded with the observed consistency of overt vocal rehearsal during instruction. Participants who executed the cued vocal sequence with high precision (e.g., P1, P6, and P7) exhibited high accuracy (100%) during character-identification probes. Conversely, variable performance observed among other participants (e.g., P2, P4, and P8) was characterized by less consistent rehearsal execution during training trials, corresponding with decreased accuracy during extended temporal delays.

Furthermore, marked individual variability was observed during follow-up phases. While several participants maintained high levels of responding, notable performance decays were observed in specific individuals (e.g., P3 and P4 dropping to 0% in certain story-recall maintenance probes). The performance variation observed in Participants 1–4 during initial post-instruction probes also highlights the impact of environmental and scheduling parameters. Due to administrative scheduling conflicts, P1–P4 experienced a 2-week delay between reaching mastery and post-instruction probe administration, compared to the standard 1-week interval maintained for P5–P9. This extended gap corresponded with temporary disruptions in independent responding when active instruction and dense schedules of reinforcement were suspended.

These individual variations highlight critical clinical considerations: instructors must carefully monitor execution consistency during acquisition and incorporate thin schedules of programmed reinforcement to support response maintenance across temporal gaps. Future research should include formal component analyses and direct classroom generalization probes to isolate active intervention components and evaluate broader ecological applications.

### 4.5. Limitations and Future Research

*Component Analysis and Experimental Control.* Despite the positive outcomes observed across participants, several methodological limitations must be acknowledged. First, because the independent variable was evaluated strictly as an integrated, multi-component instructional package, a formal component analysis was not performed. The current experimental arrangement simultaneously introduced vocal prompting, required self-echoic behavior, a constant time-delay fading protocol, a cumulative forward-chaining sequence, and programmed reinforcement alongside the cued vocal rehearsal sequence. Consequently, it is not possible to isolate the specific active component responsible for skill acquisition or the performance on untaught reverse tasks. Specifically, whether the observed variations in verbal responding were primarily associated with the explicit vocal rehearsal sequence itself, or whether they were an effect of the dense schedule of reinforcement and the structured error-correction protocol, remains undetermined.

More critically, regarding experimental control over the secondary dependent variable, the unreinforced probes for untaught character identification were not repeatedly assessed across an extended baseline. Following the logic of the concurrent multiple-baseline design, the staggered introduction of the intervention allows for the observation of corresponding changes in the primary dependent variable (story recall), but the stability and trend of the emergent character identification relation prior to intervention cannot be adequately evaluated. Furthermore, some participants demonstrated correct responding on this secondary measure during the pre-instruction probe, reducing the strength of evidence that these relations were emergent solely as a function of the primary intervention (Cariveau et al., 2022). Therefore, while the findings suggest the potential emergence of bidirectional relations following listener-style auditory–vocal arrangements, these data must be interpreted with substantial caution. Assertions regarding the standalone role of rehearsal in supporting these verbal responses must be tempered, and future research should employ systematic component or parametric analyses to examine the relative contributions and independent functional control exerted by these distinct instructional elements.

*Stimulus Generality and Narrative Heterogeneity*. Second, the reliance on a single narrative stimulus inherently limits the external validity and stimulus generality of the current findings. Because all participants were evaluated using an identical story structure, it remains unclear whether the observed outcomes reflect a generalized story-recall performance or the acquisition of a specific, topography-bound verbal chain. This limitation is relevant given the heterogeneous baseline repertoires within the participant sample. Without varying the narrative stimuli, it is not possible to determine whether the lower accuracy observed in certain participants was a function of lower rehearsal consistency or an effect of the specific thematic or syntactic complexity of the single baseline narrative. To evaluate stimulus and response generality, future inquiries should replicate these cued rehearsal protocols across a wider matrix of narrative lengths, varying both the structural complexities of the intraverbal chains and the thematic contents of the story materials.

*Direct Measurement of Stimulus and Response Generalization*. Third, although descriptive reports from teachers and family members suggested preliminary occurrences of strategy transfer, this study did not conduct systematic, direct-observation generalization probes across novel instructors, non-classroom settings, or untaught narratives. Because these collateral observations rely on retrospective verbal reports rather than continuous, direct behavioral measurement, the empirical verification of generalized verbal responding across settings remains unverified. Furthermore, the lack of an active control condition within generalization settings makes it impossible to isolate whether these reported collateral behaviors were driven by the instructional package or by extraneous environmental variables. Future research should embed unreinforced generalization probes at baseline and post-instruction milestones across diverse stimulus matrices to evaluate the cross-setting, cross-instructor, and cross-topography expansion of this taught verbal routine.

*Comparative Methodologies and the Overt-to-Covert Transition*. Fourth, future comparative investigations should feature a direct experimental comparison between purely auditory–vocal rehearsal protocols and traditional visual-aided frameworks (e.g., Valentino et al., 2015). Such research would provide empirical data regarding comparative instructional efficiency, error profiles, and long-term maintenance of each modality, particularly considering issues of restricted stimulus control and overshadowing in compound auditory arrangements (Cariveau et al., 2022). Additionally, future studies should investigate the transition from overt to covert verbal behavior (Schlinger & Blakely, 2026). Because the current study limited its continuous measurement to observable, cued vocal topographies, the exact progression from overt verbal repetition to covert self-directed responding remains an interpretive account. To advance from interpretation to direct experimental analysis, future protocols should incorporate systematic prompt-fading procedures designed to gradually attenuate the volume of vocalizations, alongside experimental dual-task or behavioral blocking paradigms. These arrangements are required to objectively evaluate how these trained overt behavioral strategies function when vocalizations recede below the threshold of direct observability.

*Participant Heterogeneity and Baseline Variance*. Fifth, the current investigation features a high degree of variance in baseline verbal repertoires and learner characteristics across the nine participants. In single-case experimental research, individual variance is managed by utilizing each participant as their own experimental control across dense, repeated measurement phases. Although the replicated, time-lagged intervention effects across participants demonstrated adequate internal validity within the observed conditions, the variance in baseline verbal skills represents a limitation regarding external generalizability. It remains experimentally unverified to what extent these idiosyncratic baseline profiles modulated acquisition velocity, error patterns, or the performance on untaught reverse tasks (Zhou et al., 2025). Consequently, the reported outcomes must be interpreted within the boundaries of this heterogeneous sample. To refine the predictive validity of this framework, future researchers should incorporate comprehensive descriptive baseline language assessments alongside larger cohorts, or employ matched-pairs arrangements within single-case designs to evaluate how specific baseline verbal thresholds interact with the acquisition of cued vocal rehearsal routines.

## 5. Conclusions

In conclusion, the current study indicates that an auditory–vocal, rehearsal-based instructional package describes a practical arrangement for establishing extended intraverbal story-recall chains in certain participants without relying on permanent external visual prompts. Examined within an operant account of verbal behavior, the outcomes suggest that establishing a cued vocal rehearsal sequence may support multiple stimulus control arrangements that correspond with high accuracy on untaught reverse tasks. By focusing on the consistency of vocal routines, this model outlines a systematic procedure for evaluating verbal skills that support independent responding within inclusive general education settings.

## Figures and Tables

**Table 1:** Participants’ information.

Participant	Age	Gender	VB-MAPP	IQ	Grade
P1 Xuan	6 y & 3 m	M	129.5	114	Preschool
P2 Nuan	7 y	M	127.5	82	First
P3 Tuan	5 y & 9 m	M	138.5	107	Preschool
P4 Dou	5 y & 2 m	M	115	102	Preschool
P5 Jiang	8 y & 7 m	M	138.5	109	Second
P6 Yu	6 y & 7 m	F	103.5	N/A	Preschool
P7 Wang	9 y & 7 m	F	102.5	43	Third *
P8 Lu	9 y & 4 m	M	107	40	Third *
P9 Yang	6 y & 4 m	M	130	92	First

* P7 and * P8 were school-aged students enrolled in the third grade of special education schools.

**Table 2:** Divergent multiple controlled intraverbal relation (story recalling, SR) and convergent multiple controlled intraverbal relation (character identification, CI).

Divergent Control Intraverbal Relation	Compound Verbal Stimuli: Four Components of Verbal Antecedents of Each 3 Characters	Convergent Control Intraverbal Relations
Ant Q:What did Ant do? Response: Ant…	1. asked what to do with the pile of grains they found	Who is this? Response: This is Ant
	2. plowed and watered the grains	
	3. participated in a race while concealing itself	
	4. obtained the harvest	
Fox Q:What did Fox do? Response: Fox…	1. suggested to plant the grains they found	Who is this? Response: This is Fox
	2. took a nap during work	
	3. proposed to have a race	
	4. won as the first to reach the finish line	
Turtle Q: What did Turtle do? Response: Turtle…	1. suggested to share the grains equally	Who is this? Response: This is Turtle
	2. planted and weeded	
	3. lost the race as the last one to reach the finish line.	
	4. shared the harvest with Ant	

**Table 3:** Mean and Range of Correct Story Recall and Character Identification Responses Across Assessment Phases.

Participant	Pre-Instruction Probes		Number of Sessions to Criteria			Post-Instruction		Maintenance Probes 2-Week		Maintenance Probes 4-Week	
	SR	CI	Ant	Fox	Turtle	SR	CI	SR	CI	SR	CI
P1 Xuan	0.00%	22.20%	4	5	4	77.8% #	100.0% #	88.90%	100.00%	77.80%	100.00%
P2 Nuan	0.00%	0.00%	5	4	4	66.7% #	44.4% #	55.60%	100.00%	66.70%	100.00%
P3 Tuan	0.00%	22.20%	4	8	4	11.1% #	88.9% #	0.00%	66.70%	0.00%	88.90%
P4 Dou	0.00%	11.10%	5	5	5	0.0% #	55.6% #	0.00%	55.60%	55.6% *	66.7% *
P5 Jiang	0.00%	22.20%	3	4	3	100.00%	100.00%	100.00%	100.00%	88.90%	88.9% *
P6 Yu	0.00%	22.20%	3	3	3	100.00%	100.00%	100.00%	100.00%	100.00%	100.00%
P7 Wang	0.00%	0.00%	6	5	5	100.00%	100.00%	100.00%	100.00%	100.00%	100.00%
P8 Lu	0.00%	0.00%	6	5	4	100.00%	66.70%	100.00%	66.70%	88.90%	55.60%
P9 Yang	0.00%	33.30%	5	5	7	100.00%	88.90%	100.00%	100.00%	100.00%	100.00%
Mean	0.00%	14.80%	4.6	4.9	4.3	72.80%	82.70%	71.60%	87.70%	75.30%	88.90%
Range	0.0–0.0%	0.0–33.3%	3–6	3–8	3–7	0.0–100.0%	44.4–100.0%	0.0–100.0%	55.6–100.0%	0.0–100.0%	55.6–100.0%

Note. SR = Story Recall; CI = Character Identification. # For P1–P4, post-instruction probes were conducted two weeks after reaching the mastery criterion due to school scheduling. * For P4 and P5, the second maintenance assessment was conducted three weeks post-instruction due to participant health issues.

**Table 4:** Quantitively summary of social validity assessment.

Evaluator Cohort	Metric Evaluated	Mean Score (M)	Standard Deviation (SD)	Score Range (Min–Max)
Language Teachers (*n* = 35)	Overall Scale Rating	4.37	0.38	3.75–5.00
	Item 4: Cultural Alignment	4.62	0.49	4.00–5.00
	Item 3: Auditory Modality Acceptability	4.54	0.51	3.00–5.00
Family Members (*n* = 20)	Overall Scale Rating	4.52	0.35	4.00–5.00
	Item 1: Social Importance of Narrative Comprehension	4.80	0.41	4.00–5.00
	Item 5: Satisfaction with Instructional Efficiency	4.45	0.50	3.00–5.00

## Data Availability

The data that support the findings of this study are available from the corresponding author upon reasonable request.

## Appendix A

*Story used for the study*

Three Friends

Once upon a time, three buddies—Fox, Turtle, and Ant—went on a journey together. While walking, they found a pile of grains on the road.

The friends stopped and looked at the grains. Ant asked, “What should we do with the grains?” Turtle suggested, “Let’s share the grains equally.” Fox had another idea, “Let’s plant them in the ground and share the harvest later. That way, we’ll have even more!” Ant and Turtle liked this plan.

Under a big mountain, they found good land. Ant plowed and watered. Turtle planted and weeded. Both worked hard, but Fox got worried. “What if the mountain falls? You two keep working. I will climb the hill to check it.” However, Fox took a nap during work.

By evening, when Ant finished plowing and Turtle finished planting, Fox came back and said, “I just saved the mountain from falling. If not, we’d lose the land and our lives.”

In autumn, Ant and Turtle harvested, and lazy Fox appeared. Fox said, “The harvest is not enough. Let’s have a race. The winner takes all. What do you think?” “How do we race?” asked Ant. Fox explained, “We’ll run from that tree. The harvest is finish line. Whoever reaches finish line first wins.”

Ant agreed, and Turtle nodded. The friends stood under the tree. “Get ready…” Fox said. Ant and Turtle got set, and Fox shouted, “Go!” as it ran. Ant concealed itself in Fox’s tail without Fox’s notice. When Fox reached the finish, Fox used its tail to hit the harvest and shouted, “All the harvest is mine! I am the first to reach the finish line!”

“Move your tail, Fox,” Ant jumped off and said, “The harvest is mine. I got here first!” Fox got mad, "What trick are you playing? You somehow got in front of me. I can’t believe you could run faster!”

Later, Turtle slowly crawled to the finish line. Turtle lost the race as the last to reach the finish line. Turtle said “Hunting dogs are looking for you. They asked me, and I said I didn’t know. They got angry and beat me up, making me late. They’re almost here. Listen, their footsteps are getting louder!”

Hearing the barking, Fox got scared and ran away. Ant obtained the harvest and shared the harvest with Turtle happily.
